# Towards rational design and optimization of near-field enhancement and spectral tunability of hybrid core-shell plasmonic nanoprobes

**DOI:** 10.1038/s41598-019-52418-9

**Published:** 2019-11-05

**Authors:** Debadrita Paria, Chi Zhang, Ishan Barman

**Affiliations:** 1 Department of Mechanical Engineering, Johns Hopkins University, Baltimore, MD USA; 2 Department of Oncology, Johns Hopkins University School of Medicine, Baltimore, MD USA; 3 Department of Radiology & Radiological Science, Johns Hopkins University School of Medicine, Baltimore, MD USA

**Keywords:** Nanoscience and technology, Optics and photonics

## Abstract

In biology, sensing is a major driver of discovery. A principal challenge is to create a palette of probes that offer near single-molecule sensitivity and simultaneously enable multiplexed sensing and imaging in the “tissue-transparent” near-infrared region. Surface-enhanced Raman scattering and metal-enhanced fluorescence have shown substantial promise in addressing this need. Here, we theorize a rational design and optimization strategy to generate nanostructured probes that combine distinct plasmonic materials sandwiching a dielectric layer in a multilayer core shell configuration. The lower energy resonance peak in this multi-resonant construct is found to be highly tunable from visible to the near-IR region. Such a configuration also allows substantially higher near-field enhancement, compared to a classical core-shell nanoparticle that possesses a single metallic shell, by exploiting the differential coupling between the two core-shell interfaces. Combining such structures in a dimer configuration, which remains largely unexplored at this time, offers significant opportunities not only for near-field enhancement but also for multiplexed sensing via the (otherwise unavailable) higher order resonance modes. Together, these theoretical calculations open the door for employing such hybrid multi-layered structures, which combine facile spectral tunability with ultrahigh sensitivity, for biomolecular sensing.

## Introduction

Nanoparticle represent promising platforms for biomolecular detection and imaging since they have the ability to modulate pharmacokinetics through surface functionalization, to integrate various physiochemical detection in a single probe by leveraging various properties (for e.g. plasmon resonance, temperature/PH responsivity or magnetization) and to enhance the weak imaging or sensing signal arising from a biological system. Considerable effort has been directed towards the design of more sensitive and efficient metal nanoparticles for biosensing^1–3^ and imaging^4^. For instance, several novel detection schemes based on magneto-plasmonics^5,6^ and metallic thin films^7,8^ have been demonstrated. Of particular interest in the sensing domain and relevant to our current study is the capability of these metal structures in enhancing the electromagnetic field in its vicinity, which can sense minute perturbations in the surrounding and also amplify the weak Raman signal arising from a biological sample or dilute analytes (surface enhanced Raman scattering (SERS))^9^. These nanoprobes have also been utilized to enhance the weak fluorescence signal from a labelled mouse tumor *in vivo*^10^- by exploiting metal enhanced fluorescence (MEF)^11,12^.

The amplification observed in a typical SERS signal can be attributed to the combination of chemical enhancement (CE), arising from interaction between metal and analyte molecule, and electromagnetic enhancement (EE), arising from the ability of the metal nanostructure to enhance the local field. Since the EE is significantly larger compared to the CE, there has been a considerable effort to develop systems that offer intense field localization (through creation of high-density “hotspots”) often using a cluster of metallic nanoparticles^13,14^. Such enhancement, even though has been used to detect single molecule^15^, may however be challenging to control and reproduce with undefined spatial positions of the hotspots. The inability to reproduce the enhancement has led to the theorization of the “SERS-uncertainty principle”^16^. Addressing this critical drawback requires the designing of metallic nanoprobes with well-defined and predictable nearfield enhancement (NE) with known spectral resonance energies. Further, in the context of MEF detection, knowing the exact spatial distribution of the hotspots can aid in placing the fluorescence tag in the exact position such that the fluorescence signal can be maximized. Additionally, the wavelength of the resonance of these nanoprobes can be tailored to match the emission of a fluorophore.

Various approaches have been explored to improve SERS and MEF sensitivity, many of which revolve around the central idea of fabricating structures that are resonance-matched with the analyte and maximize the nearfield enhancement. Several studies have adopted a core shell (CS) design, comprised of a dielectric sphere covered by a metallic shell^17^ or vice versa^18^, to address this issue. A CS structure allows more flexibility in the choice of resonance position apart from providing better field amplification compared to a solid metallic nanoparticle. Building on the central design principles of the core-shell architecture, which has been successfully exploited in biosensing, drug delivery and imaging^19^, multi-layer core-shell structures that are composed of a metallic shell and core separated by a dielectric spacer have been lately proposed^20,21^.

Although a core shell structure has been demonstrated to be a suitable candidate for various applications like photothermal cancer therapy^22,23^, sensing^24^, catalysis^25^, bioimaging^23^ etc., a multilayer core shell (MCS) structure^20,21^, composed of more than one shell layer and a metallic core, provides several advantages. For example, higher order modes are prominent in MCS structures leading to appearance of multiple resonances - typically one in the near IR region, which is absent in CS. This allows fresh avenue for multiplexed biomolecular sensing in complex matrices. Also, interaction of the metallic core with different modes of the metallic shell allows possibility for greater spectral tunability. Using MCS structures, similar optical characteristics as a CS structure can be achieved in smaller sizes that would allow better targeting ability and pharmacokinetics *in vivo*, as larger probes often face significant hindrances in reaching their targeted location^26^.

Prior studies on MCS structures by Nordlander, Halas and co-workers have focused on the “plasmon hybridization model”, which is an analog of the molecular orbital theory for describing the plasmon response^27–29^ in a concentric shell structures. Very recently, experimental demonstrations of the optical properties of the multilayer nano-shell structures have been shown by the same group^30–32^. Furthermore, Kodali *et al*. have theoretically demonstrated the benefits of employing MCS structures for SERS measurements^33^, and a recent study by Ayala-Orozco *et al*. has reported the embedding of fluorescence tags in such structures for MEF-type detection^34^. Yet, most of the theoretical studies of MCS have focused on its optical properties. Although extensive literature exists for the assessment of plasmonic nanoparticles for suitability as a SERS probe, there has been very little focus for MCS constructs on a systematic study of the parameters for the tunability of its resonance peak^35^ and optimization of its near field enhancement (NE)^21,33^. Moreover, the possibility of leveraging a dimer architecture composed of two MCS structures, which we reason can boost the NE by several orders of magnitude, has been under-appreciated.

In this paper, we perform a detailed numerical investigation of the design parameters of hybrid MCS nanoprobes that are composed of silver core-dielectric shell-gold shell (HMCS), and their impact on tunability of the resonance peak in the near IR region as well as optimization of the NE. Our choice for this configuration is driven by a combination of efficient electromagnetic field enhancement and biomedical suitability considerations. In particular, silver provides more field enhancement, hence is chosen as the inner core material, but is prone to degradation and oxidation, thus, gold is chosen as the shell material instead. For complex SPR coupling such as in a HMCS structure, determination of NE via analytical methods is extremely challenging; hence, we resort to the use of finite element analysis to understand their plasmonic resonance properties. We first compared HMCS structure with a solid metallic particle and a conventional core shell in terms of their spectral characteristics and NE and show that the HMCS structure is superior in both these aspects. Moreover, the HMCS structure shows a highly tunable resonance feature in the near IR, which is technologically relevant for sensing in the tissue-transparent diagnostic window (*ca*. 700–1100 nm). Our investigations, in this regard, led us to additionally theorize that the use of coupled structures would render considerable advantages in terms of larger near field amplification and appearance of higher order resonances. Based on our conjecture, we explored the hitherto unexplored interactions between two similar HMCS structures in a dimer configuration in terms of their spectral properties and near-field coupling. Our analysis shows that the SERS enhancement near the hotspot goes up to 10^11^, which is at the maximum limit for an achievable electromagnetic enhancement factor^36^.

The addition of further layers of metal and/or dielectric does not show substantial benefits in terms of EM enhancement^21^, which lessens the enthusiasm for undertaking complicated fabrication processes that are required for creating such structures. We envision that detailed knowledge of these theoretical underpinnings will facilitate optimized design of multi-modal, single-scaffold nanoprobes and their future application in biomolecular sensing and imaging.

## Results and Discussion

### Design and spectral characterization of HMCS structure

**Figure 1:**
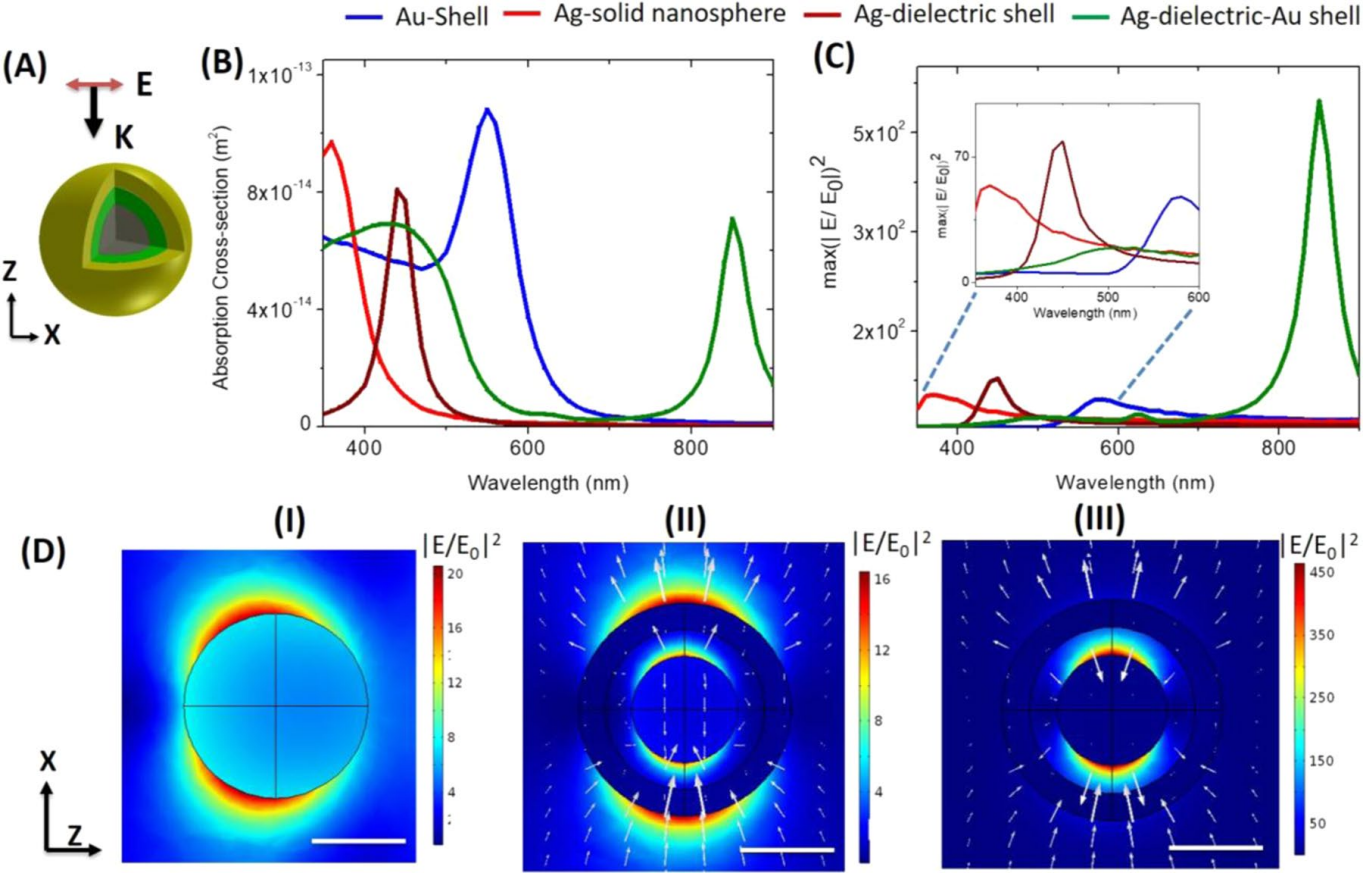
HMCS structure compared to a solid metallic sphere and core shell nanoparticles: (**A**) Scheme of simulation with the direction of propagation and polarization marked. grey center indicates silver solid sphere of diameter 40 nm, surrounded by a 10 nm thick silica layer marked in green, and 10 nm thick gold layer marked in yellow. (**B**) Absorption cross-section of HMCS structure compared to solid silver spheres, a hollow gold sphere and a silver-dielectric shell nanoparticle. (**C**) Comparison of enhancements (maximum |E/E_0_|^2^ in the entire volume) for the different cases. The inset shows the enhancement at the higher energy peak (zoomed in). (**D**) Electric field distribution plotted for solid metallic particle (I) and for the antibonding mode at 440 nm (II) and the bonding mode at 850 nm (III) of the HMCS structure. The electric field vectors for the bonding and the antibonding modes are also plotted. All the scale bars are 40 nm.

Figure 1A shows the design of a HMCS structure composed of a 40 nm (diameter) silver nanosphere covered by a dielectric shell (permittivity ɛ-3.9) and a gold shell, each having a thickness of 10 nm (the schematic of the simulation model is provided in the Supplementary Information Fig. S1). Previously, multi-layer core-shell structures that are composed of a gold core and a gold shell have been explored^20,30^. Our selection of the core and two shell materials were governed by the higher EM enhancement capabilities of silver and the superior biocompatibility of gold, respectively.The choice of dielectric material will depend on the specific application, and the reporter molecule to be functionalized. For our numerical study, a dielectric material with a constant refractive index was used. The effect of the refractive index of dielectric material as well as different plasmonic materials for the outer shell on the resonance of HMCS structure is provided in the Supplementary Information (Fig. S2).

We first compare the performance of such a HMCS structure with that of a solid metallic nanoparticle and a standard core-shell design in terms of NE and spectral characteristic. The scheme of simulation is shown in Fig. 1A. The HMCS structure repeats periodically with an edge to edge distance of 50 nm infinitely in the X and Y direction. The excitation and polarization direction of HMCS is marked in Fig. 1A. Further details of the FEM model and scheme of simulation, including the comparison of periodic and single HMCS structure (Fig. S3), is detailed in the Supplementary Information and Materials & Methods. Figure 1B compares the absorption cross-section of HMCS with a single silver nanosphere (diameter: 80 nm), a metal-core dielectric-shell nanoparticle (silver core diameter: 40 nm, dielectric shell thickness: 10 nm), and hollow gold shell (diameter: 80 nm, thickness: 10 nm). As expected, the silver solid nanoparticle shows a single resonance peak corresponding to a dipolar resonance centered around 360 nm. Adding the dielectric shell to the solid silver nanosphere red-shifts the resonance to 440 nm. This is along expected lines, too, as plasma frequency of metallic nanostructure is known to be extremely sensitive to its surrounding dielectric^37^, a phenomenon widely used for refractive index sensing^38^. Moreover, it has been previously demonstrated^17,39^ that a configuration composed of metallic shell provides greater tunability to its resonance peak over a larger region of the electromagnetic spectrum compared to that of a dielectric shell. The hollow gold shell with dielectric constant 1 assigned to the core has a strong absorption at 550 nm, red-shifted from a solid gold sphere resonance at 510 nm (comparison is provided in the Supplementary Information Fig. S4). Importantly, when another metallic layer (i.e. the gold shell) is introduced to the core shell nanoparticle, a distinct resonance peak appears in the near infra-red (IR) region^20,21^. The interaction between the metallic core and the shell leads to splitting of the peak into a hybridized bonding mode at 850 nm and an antibonding mode at 440 nm (as seen in Fig. 1B)^27–30^. The resonance positions of the HMCS structure do not correspond to the resonance of the individual components (Fig. 1B), which further confirm the fact that the resonance arises from complex interactions of the individual components.

Figure 1C compares the maximum NE of solid metallic nanoparticles with the core shell and HMCS. Here, the maximum enhancement, which occurs either at the shell surface or at the core surface, is calculated as the maximum of |E/E_0_|^2^ (where E_0_ is the incident field and E is the total field). As seen in Fig. 1C, the amount of NE is similar in case of the solid silver nanoparticle, the core-shell nanoparticle, and the gold hollow shell. In contrast, the bonding mode at the near IR region for the HMCS shows considerably stronger near field enhancement, though the enhancement at the antibonding mode is much weaker. The dipole of the inner core and the shell are oppositely aligned in case of the bonding mode leading to a strong coupling, contrary to the antibonding mode where dipoles are aligned in the same direction, giving rise to a weak interaction and a resonance peak at a higher energy^27,30^. Thus, the enhancement for the bonding mode is about an order of magnitude higher than the antibonding mode underscoring the higher sensitivity of this feature for molecular sensing applications.

In order to investigate the corresponding NE for the HMCS, we used finite element analysis tools, as the plasmon coupling of a multilayer core shell nanoparticle is a complex function of core-shell and shell-shell interaction such that the NE cannot be described by simple analytical solutions. Figure 1D plots the electric field vectors and the spatial distribution of the local field enhancement for the solid silver nanosphere (Fig. 1D(I)) and HMCS structure at 440 nm (Fig. 1D(II)) and 850 nm (Fig. 1D(III)). The electric field vector at the surface of the core and the shell are oriented in the same direction indicating alignment of the dipoles in case of the antibonding mode at 440 nm (Fig. 1D(II)), whereas, at 850 nm the electric field vectors are oriented in the opposite direction (Fig. 1D(III)) indicating strong coupling at the bonding mode. The spatial distribution of electric field indicates that as expected, the solid particle behaves as a dipole, radiating in the direction of the incident polarization (X-axis). The HMCS structure, on the other hand, has enhancement arising both at the outer surface of the gold shell and the inner dielectric spacer at 440 nm, and an enhancement confined strongly in the silica shell at 850 nm. Strong confinement of local field in the interstitial dielectric layer allows the possibility of ultra-sensitive SERS detection, and super-resolution imaging by embedding Raman reporter molecules^33^ or fluorescent tags^34^ within the layer.

### Optimization of near field enhancement and spectral tunability through variation of structural parameters of the HMCS

SERS sensing and plasmon-enhanced imaging applications rely on the perturbation of the plasmon-mediated, highly confined and amplified local field by the molecule(s) of interest. Thus, for obtaining higher sensitivity, the primary requirement is to optimize the near field enhancement. In the previous section, we have shown that the HMCS structure shows a strong resonance in the N-IR region and, hence, its promise for biosensing in the so-called “tissue-transparent” window^40^. In this section, we discuss the different dimensional parameters, which contribute to maximizing the local electric field near the HMCS structure as well as the tunability of its plasmon resonances.

#### Effect of dielectric thickness

**Figure 2:**
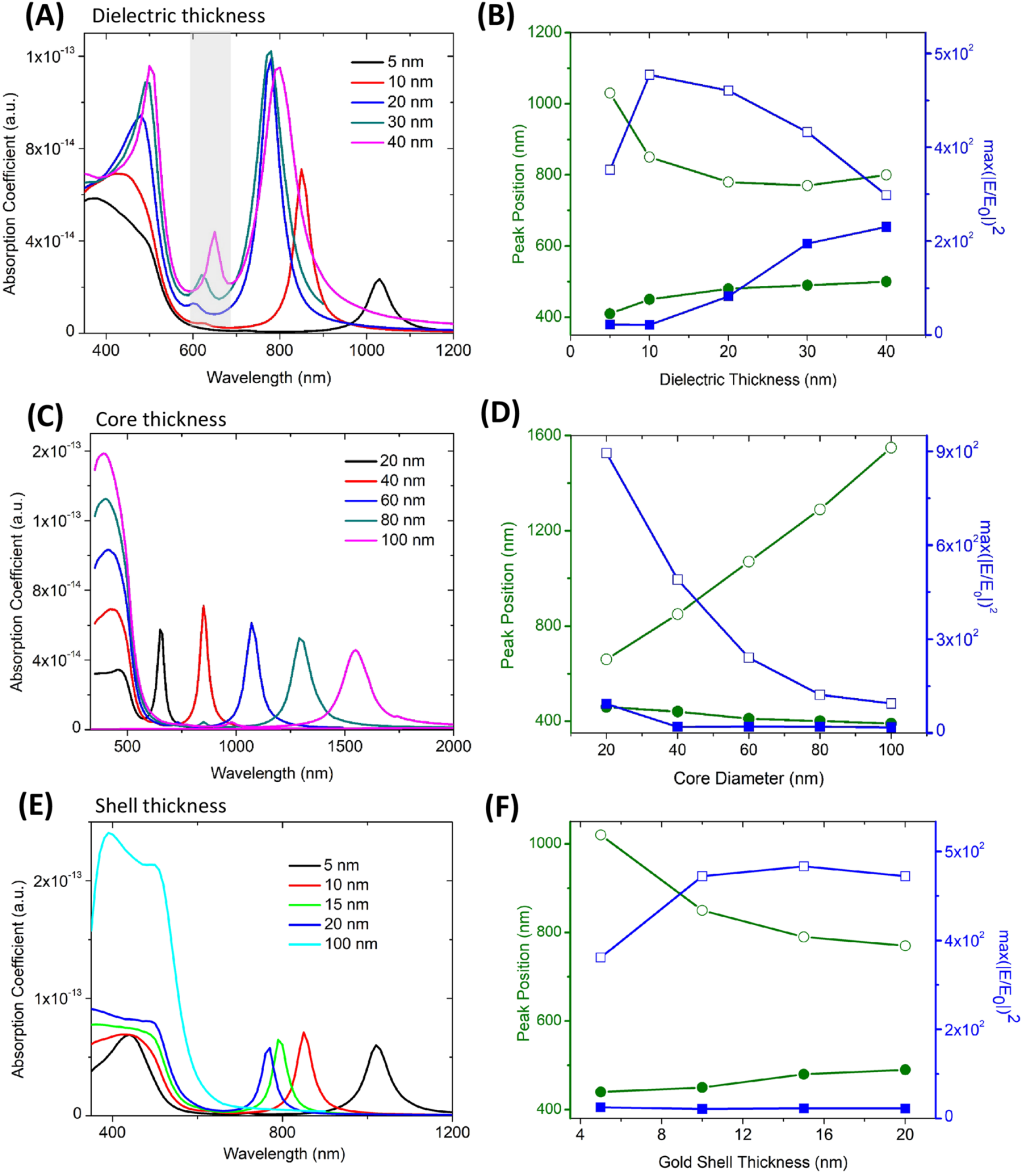
Tunability of HMCS structure based on dimensional parameters: (**A**) Absorption cross-section with variation of dielectric spacer thickness for a constant silver core and gold shell thickness of 40 nm and 10 nm respectively. The higher order mode due to increased size of HMCS structure is highlighted in the figure. (**B**) Plot of resonance positions and NE for the bonding and the antibonding mode for various dielectric thickness. (**C**) Absorption cross-section with variation of silver core for a constant dielectric and gold shell thickness of 10 nm. (**D**) Plot of resonance positions and NE for the bonding and the antibonding mode for various core dimension. (**E**) Absorption cross-section with variation of gold shell for a constant dielectric thickness and silver shell size of 10 nm and 40 nm respectively. (**F**) Plot of resonance positions and NE for the bonding and the antibonding mode for various silver shell dimension. For the figures (**B**,**D**,**F**), the green line with solid sphere represents the resonance position for the antibonding mode, while the one with open circles represents that of bonding mode. The blue line with solid squares represents NE at the antibonding resonance, while the one with open squares represents the NE at the bonding resonance.

Figure 2A plots the absorption spectra of the HMCS structure when the dielectric thickness is varied by keeping the silver core diameter fixed at 40 nm and the gold shell thickness at 10 nm. An increase in thickness of the dielectric layer leads to an accompanying increase in the gold shell diameter as well as an overall increase in the size of the HMCS structure thereby increasing the absorption cross-section. Additionally, due to the increased size of the silver shell, a third peak appears near 600 nm (highlighted band in Fig. 2A), which is a quadrupolar bonding mode^30^. The dielectric layer determines the degree of interaction between the metallic core and the shell. As the dielectric layer becomes thicker the core and the shell interact weakly, thus, blue shifting the bonding mode (initially at the N-IR region) closer to the resonance of a hollow gold shell of similar dimension (Supplementary Information Fig. S5)^27^. As can be seen from the simulation results of the bonding mode (represented by the green line with open circles in Fig. 2B), plasmon resonance shifts by *ca*. 200 nm with an increase in dielectric thickness from 5 nm to 40 nm. The antibonding mode, whose energy was close to that of a core-shell nanoparticle (silver core-gold shell, Fig. 1B), on the other hand, shows a slight red shift, as indicated by the green line with solid circles in Fig. 2B. The maximum of |E/E_0_|^2^ is also plotted in the same figure. The enhancement present at the antibonding mode (blue line with solid squares) is far less that that at the bonding mode (blue line with open squares) for thinner dielectric layers. The enhancement at the bonding mode increases initially with the dielectric thickness, due to increased size of the shell and stronger coupling between inner core and the shell but gradually decreases after a threshold thickness due to reduced interaction and decoupling of their plasmon resonances^21^. A thick dielectric layer will reduce the enhancement due to reduced interaction between the core and the shell, while a very thin dielectric layer will also diminish enhancement due to quantum mechanical effects^41^. Thus, for a fixed silver shell and gold core dimension, our findings confirm that there exists an optimum dielectric layer thickness for achieving the maximum near-field enhancement.

#### Effect of core size

Figure 2C demonstrates the tunability of resonance peaks with an increase in the silver core radius by keeping the dielectric spacer and gold shell thickness constant at 10 nm. The position of the antibonding mode remained almost constant with a minimal blue shift (Fig. 2D) towards the resonance of an isolated solid silver nanoparticle. The bonding mode, on the other hand, remarkably shifts by 890 nm into the IR region for a core diameter increase of 80 nm. An increase in core size also leads to a corresponding increase in the shell diameter, leading to the appearance of a similar quadrupolar peak as in the previous case, which is difficult to visualize in the graph due to the much stronger absorption resonance at the antibonding mode (Fig. 2D). The increase in core size has no effect on the weakly coupled core and shell at the antibonding mode leading to minimal/no effect on the enhancement (Fig. 2D). The enhancement at the bonding mode decreases with an increase in core size (Fig. 2D) with a maximum value at a core diameter of 40 nm. Thus, we find that while a resonance position further in the IR can be generated by tweaking the HMCS core size, such a design yields a diminished NE. For example, IR spectroscopic applications which require monitoring of the spectral shift^42^ can make use of larger core thicknesses, but for sensing applications that depends on NE^43^ a silver core size of about 40 nm seems better suited based on our simulation results.

#### Effect of metallic shell thickness

We further study the effect of gold shell thickness on tunability and NE with a constant core size of 40 nm and a dielectric spacer thickness of 10 nm (Fig. 2E). It can be observed that the resonance of the bonding mode can be tuned from red to the N-IR region by decreasing the gold shell thickness^17^ (Fig. 2F). At an extreme case of 100 nm gold shell thickness, we observe that the bonding mode blue shifts and merges with the antibonding mode. For a 5 nm thick gold shell, the resonance is around 1030 nm; further thinning of the metal shells leads to damping of plasmon due to reduction of electron mean free path on collision with the surface and, hence, requires a modified dielectric function of the metal^21,44^ to simulate the resonance. Thinner shell reduces the NE due to damping; on the other hand, thicker shells also reduces the enhancement due to increased absorption. Thus, from Fig. 2F, it can be deduced that, for a core size and dielectric spacer dimensions of 40 nm and 10 nm, respectively, a gold shell thickness in the range of 10–20 nm provides the maximum NE near the lower energy resonance mode.

In summary, our analysis reveals that the bonding mode is highly tunable by variation of any of the core, shell or dielectric spacer dimensions, unlike the antibonding resonance peak. Moreover, an increase in core size, while keeping other dimensions constant, permits maximum tunability of the plasmon energy at the bonding mode from red to N-IR region. Furthermore, NE is much higher at the bonding mode compared the antibonding mode, where coupling is weak due to aligned dipoles in the core and the shell. The NE at the lower energy plasmon peak can be maximized by also varying the dimensional parameters. While designing sensing probes all the above factors should be considered to achieve maximum spectral shift, maximum NE or an optimal combination of both.

### Creating a plasmonic dimer with HMCS nano-structures

A straightforward approach for increasing the NE is to place two plasmonic nanoparticles in close proximity in a configuration known as a “plasmonic dimer”. Such a construct comprised of two solid nanoparticles has been studied theoretically^29,45^. Demonstrations using a dimer configuration for sensing have also been reported^46,47^, which confirm that the NE of such dimer structures is several times higher than that of an isolated nanoparticle^46^. Yet, despite the potential utility of such a design, core-shell nanostructures have not been employed in this configuration to the best of our knowledge. Here, we reason that, a dimer composed of HMCS will not only provide higher NE but also suitable spectral tunability.

**Figure 3:**
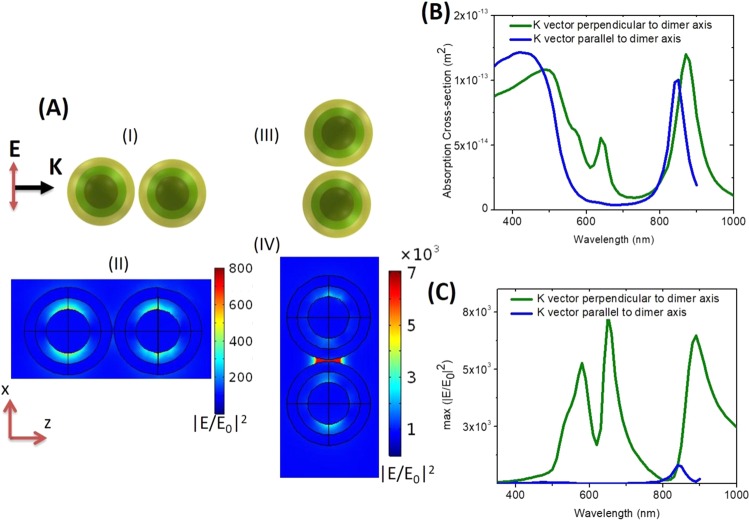
Polarization dependence: (**A**) Schematic of two different configurations where the polarization of incident field is perpendicular to the dimer axis (I) and parallel to the dimer axis (III). (II) The spatial distribution of electric field corresponding to the configuration (I). (IV) spatial distribution of electric field corresponding to the configuration (III). (**B**) Compares the absorption cross section for the two configurations in (A)(I) and (III). (**C**) Plots the maximum of |E/E_0_|^2^.

The direction of polarization of the incident field with respect to a dimer orientation is an important parameter for coupling between the two HMCS structures. Figure 3A plots the schematic and spatial distribution of the local electric field enhancement for two polarization conditions, which we have numerically explored for the HMCS dimer composed of a silver core - dielectric layer - gold shell with dimension of 40 nm, 10 nm and 10 nm respectively, and interparticle spacing of 2 nm. Figure 3A(I) shows the scheme of simulation where the polarization direction is perpendicular to the dimer axis and its corresponding distribution of electric field enhancement at the lowest energy resonance peak is plotted in Fig. 3A(II). It can be clearly observed that the two HMCS structures do not couple, and the enhanced field is mostly confined in the dielectric layer. On the other hand, when the polarization is parallel to the dimer axis (Fig. 3A(III)), the two HMCS structures couple strongly leading to an extremely amplified field at the inter dimer spacing (Fig. 3A(IV)). This is an interesting finding, owing to the complete lack of prior knowledge on the existence and the spatial localization of “hotspots” in core-shell-based dimer constructs. The field enhancement and localization are stronger in the interparticle space as compared to that in the spacer layer of the individual HMCS structure. Our observation suggests that such a configuration maybe beneficial in a situation, where integrating the reporter molecule in the HMCS structure is challenging.

The absorption cross-section for both the polarization conditions are plotted in Fig. 3B. When the polarization direction is parallel to the dimer axis, resonances similar to the bonding and the antibonding modes of isolated HMCS structures exist, but peak positions are slightly red shifted due to interactions between the two HMCS structures. Interestingly, a hitherto unseen peak appears at 640 nm due to a strong dipolar coupling between the two HMCS structures. Such a peak is absent in the HMCS dimer when the polarization direction is perpendicular to the dimer axis leading to the absence of plasmon interactions between the two particles; hence, the resonance peak closely follows that of an isolated HMCS structure. The maximum field enhancement plotted in Fig. 3C, also, reconfirms the required polarization of incident field for maximizing NE.

**Figure 4:**
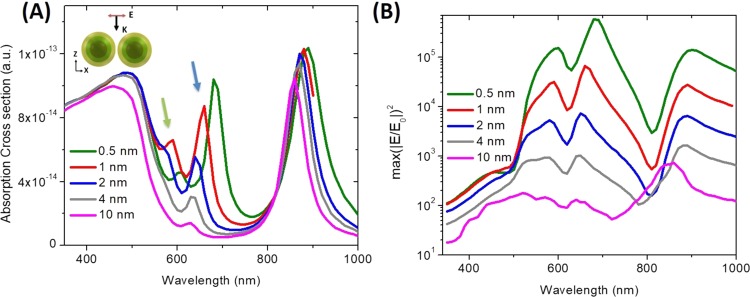
Dependence on interparticle spacing: (**A**) Plots the absorption spectra of a HMCS dimer with variation in interparticle spacing. The resonance peak due to the dipolar and quadrupolar coupling between the two HMCS structures is indicated with a blue and a green arrow respectively (for an interdimer spacing of 1 nm). (**B**) The surface integral of enhancement in the XY plane plotted across wavelengths for various interparticle spacing. Significant enhancement is present for all the modes except the antibonding mode with reduction in distance between the two HMCS.

In order to exploit the strong coupling and intense field confinement in a dimer configuration, it is important to consider the inter-dimer spacing. Figure 4A plots the absorption cross-section of HMCS dimer of similar dimension as before for varying interparticle spacing. The dipolar coupling peak near the red region (marked in the figure with a blue arrow for an interparticle spacing of 1 nm) becomes larger and red shifted with reduction in interparticle spacing indicating increased interaction. The quadrupolar coupling peak (marked in the figure with a green arrow for an interparticle spacing of 1 nm) also appears and becomes prominent in the red region with the reduction in interparticle distance. The maximum NE is plotted as a function of wavelength for various interparticle distance in Fig. 4B. As the interparticle spacing reduces, the NE near the dipolar coupling peak becomes larger than that near the bonding mode. Moreover, significant enhancement is observed for all the modes except the higher energy antibonding mode. It is however important to note that further reduction of interparticle spacing may not lead to higher enhancement, since at a sub nanometer length scale quantum tunneling^48,49^ and non-local effects^50–52^ may influence the enhancement. In our numerical simulation these effects were not considered and thus, the field enhancement values and resonance conditions for the sub-nanometer spacing is an approximation indicative of the general trend with reduction of interparticle spacing.

Overall, the HMCS structures exhibit strong coupling behavior when the incident polarization is aligned with the dimer axis, similar to a dimer composed of two solid nanoparticles. More importantly, in a dimer composed of HMCS structures, several higher order resonance modes become prominent as a result of complex interactions between the two HMCS structures (i.e. the shell and the core), which is not visible in a dimer composed of solid nanoparticles. This shows promise as the basis of a multi resonance system with different degree of tunability of individual peak for multiplexed measurements^53^. Our calculations reveal that the maximum NE is greater than 10^5^ and can enable SERS enhancements in the range of 10^11^ (where the enhancement factor for SERS measurements is given by the 4^th^ power of the enhancement of the electric field amplitude) - which represents the maximum theoretical limit for plasmon-enhanced Raman scattering^36^. An important point to note is that although the reduction of interparticle spacing can lead to higher enhancement in HMCS structure, achieving a sub nanometer interparticle spacing is experimentally challenging. With the advancement in nanotechnology, fabricating a narrow plasmonic hotspot is not impossible as there have been several recent reports on demonstration of sub-nanometer spacing using a single layer graphene sheet as a spacer^47,54,55^ or DNA origami for controlling the spacing and integration of reporter molecule in the narrow gap^56^.

## Conclusion

Maximizing the near field enhancement and tunability of the resonance peak in the near IR simultaneously represent a major challenge underlying nanoparticle-enhanced imaging measurement in biomedicine, ranging from fields as diverse as hepatology, neurobiology and oncology. Our current study seeks to systematically investigate design parameters that can address this classical trade-off and guide the architecture of hybrid multilayer core shell nanoparticle. Significantly expanding on the idea of multi-layer probe designs, we propose the use of distinct plasmonic nanomaterials sandwiching a dielectric layer to form a hybrid structure that shows improved near-field enhancement and substantially better spectral tunability in the visible to near-IR region in comparison to a solid or a classical core-shell plasmonic nanoparticle. Owing in part to their spherical shape, such multilayer nanoparticles can be readily fabricated using well-established chemical synthesis protocols. Furthermore, our simulations reveal that, in addition to providing very high near-field enhancement, dimers of such structures offer several higher order resonance modes that can be harnessed for multiplexed biomolecular sensing. Numerical simulation reveals the precise location of the hotspots, i.e. either in the dielectric layer or the interparticle space and will enable precise placement of reporter or analyte molecule for higher sensitivity. The outer gold layer is chemically inert and is not prone to oxidation or reaction with the environment ensuring the stability and bio-compatibility of the nanoprobe. Also, outer gold layer can be readily bioconjugated to a diverse set of ligands, this present work provides effective insights that can lead to the adoption and subsequent translation of such nanoparticles for MEF and SERS-based molecular detection and imaging in biological tissue.

## Materials and Methods

Our numerical simulation was done using finite element method-based software (COMSOL Inc., Burlington, MA, USA). The HMCS structures were arranged in a square lattice. In order to simulate an infinite array in the X and Y directions, a periodic boundary condition is considered. An input port in the Z direction excites the array. As a boundary condition at the input and the output port, a perfectly matched layer is applied. Tetrahedral mesh element size of 10 nm for the nanostructure and λ/6 for the rest is applied respectively. The dielectric constant of the spacer is kept constant whereas the metals were considered to have wavelength dependent dielectric values (reference for the values are provided in the supporting information).

## Supplementary information

Supplementary file
