# Supplementary material for: Towards rational design and optimization of near-field enhancement and spectral tunability of hybrid core-shell plasmonic nanoprobes
Source: Sci Rep. 2019 Nov 5;9:16071. doi: 10.1038/s41598-019-52418-9 (PMC6831636; doi:10.1038/s41598-019-52418-9)
Supplement: Supplementary file 1 — Supplementary file [file 41598_2019_52418_MOESM1_ESM.docx]

**SUPPLEMENTARY INFORMATION**

**Towards rational design and optimization of near-field enhancement and spectral tunability of hybrid core-shell plasmonic nanoprobes**

Debadrita Paria**^1^,** Chi Zhang**^1^**, Ishan Barman**^1, 2, 3*^**

^1^Department of Mechanical Engineering, Johns Hopkins University, Baltimore, MD, USA.

^2^Department of Oncology, Johns Hopkins University School of Medicine, Baltimore, MD, USA. ^3^Department of Radiology & Radiological Science, Johns Hopkins University School of Medicine, Baltimore, MD, USA

**Keywords:**  Plasmonics, multilayer core-shell, near field enhancement, SERS, fluorescence imaging, FEM

***Correspondence:**

Ishan Barman

Johns Hopkins University

Whiting School of Engineering

Department of Mechanical Engineering

Latrobe Hall 103

Baltimore, MD 21218, USA.

Office Phone: 410-516-0656

E-mail: ibarman@jhu.edu

**Conflict of Interest:** The authors disclose no potential conflicts of interest.

***Details of simulation:***

The simulation model is shown by the schematic given in Fig. S1. The periodic boundary condition was applied in both X and Y direction to simulate an infinitely repeating HMCS structure in both directions. Frequency-dependent dielectric constants of silver[^1^](https://paperpile.com/c/mVBBkg/3qUZ), copper[^2^](https://paperpile.com/c/mVBBkg/cwXR), gold[^2^](https://paperpile.com/c/mVBBkg/cwXR), platinum[^3^](https://paperpile.com/c/mVBBkg/Z79X) and aluminium[^3^](https://paperpile.com/c/mVBBkg/Z79X) were used in the simulation from the literature.


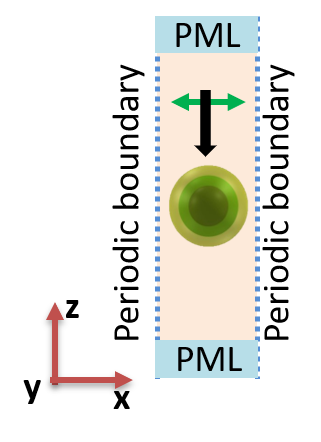


*Figure S1: Simulation model. The green arrow shows the polarization of the incident field. The black arrow indicates the propagation direction.*

***Variation in near-field EM enhancement as a function of plasmonic material:***

We explored the effect of varying the dielectric constant on the NE of a HMCS structure. Fig. S2 A plots the maximum NE in HMCS structure when the permittivity (ɛ) of the dielectric spacer is varied. Initially, with the increase in ɛ (from 1 to 2.89) the NE of the bonding mode increases. However, at a higher ɛ due to dielectric screening[^4^](https://paperpile.com/c/mVBBkg/Msmb), the coupling between the inner core and the outer shell decreases leading to a reduction in NE.

Fig. S2B re-plots the maximum NE when the shell is replaced by different noble metals in the HMCS structure by fixing the core material as silver and interlayer dielectric with ɛ of 3.9. A silver shell layer provides the maximum NE followed by gold and copper. The NE for platinum and aluminum, expectedly, is orders of magnitude less. Even though silver shell shows the maximum amount of enhancement, a nanoprobe with a silver surface can pose several problems including oxidation, instability and toxicity particularly for biosensing or imaging applications. Taken together, these findings re-affirm that the HMCS structure with a gold shell offers the most suitable candidate for such applications.


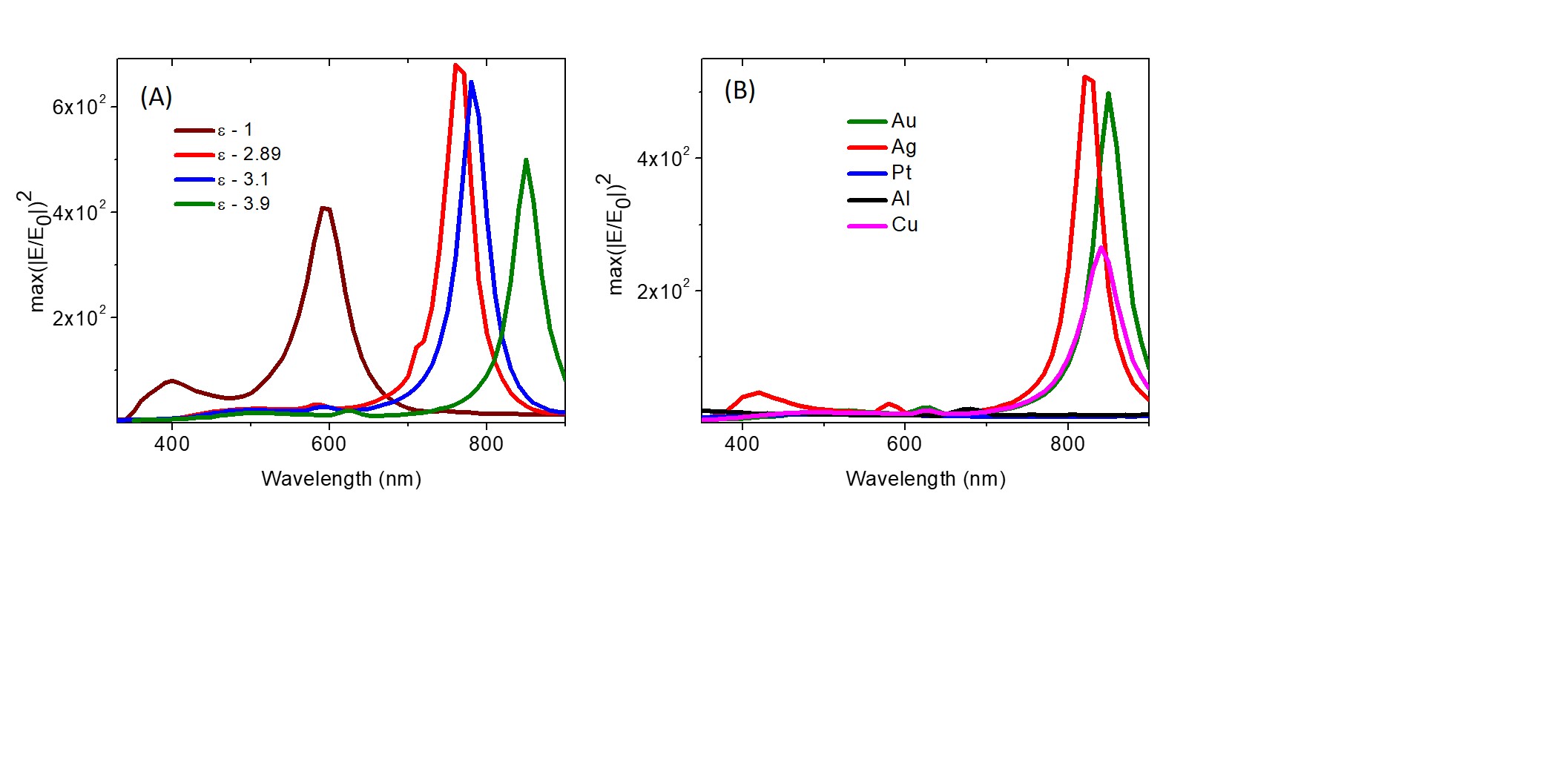


*Figure S2: (A) Maximum NE across various wavelengths for a HMCS structure (40 nm silver core-10 nm dielectric layer-10 nm gold shell) for various permittivities (ɛ) of the dielectric spacer layer. (B) Maximum NE across wavelength for a HMCS structure(40 nm silver core-10 nm dielectric layer(ɛ-3.9) -10 nm noble metal shell) for various noble metals as the shell material.*

***Periodic system vs single nanoparticle***


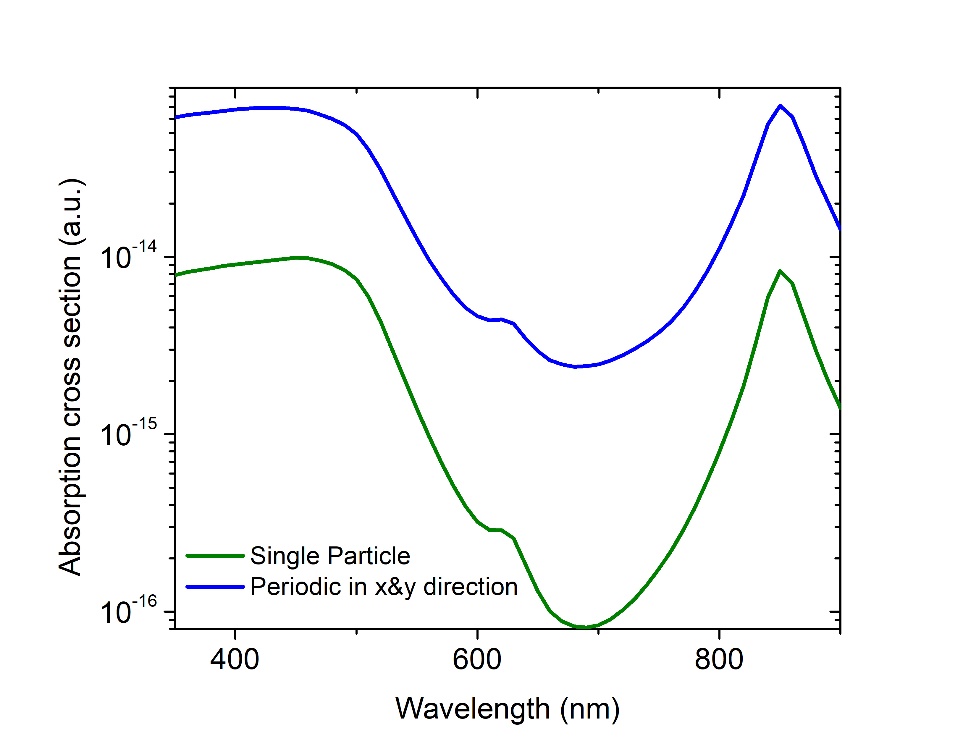


*Figure S3: Comparison of absorption cross section of an infinitely repeating (x and y direction) nanoparticle (silver core -dielectric and gold shell) with a single nanoparticle particle of same dimension.*

***Resonance of a gold solid sphere vis-à-vis a gold hollow shell***


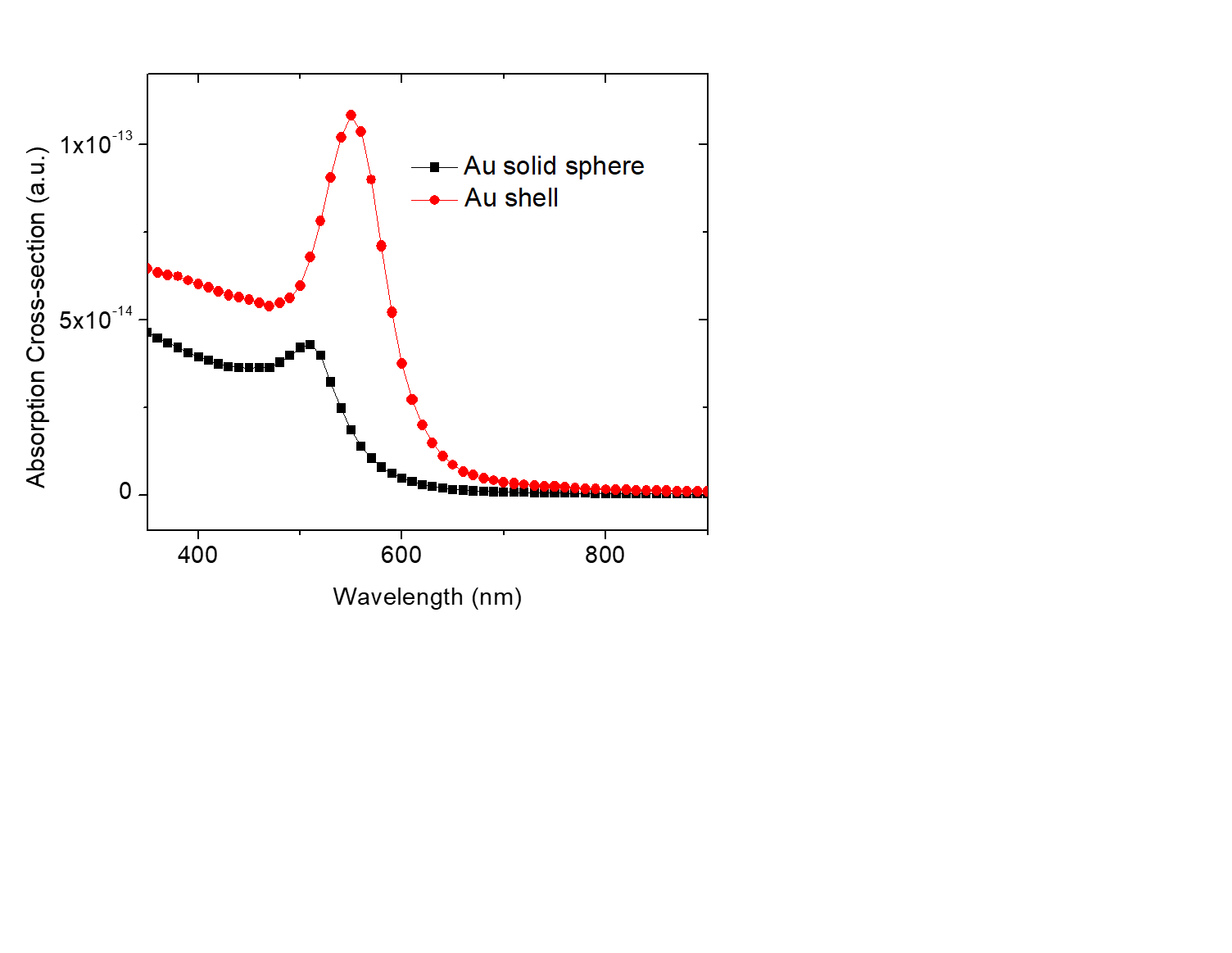


*Figure S4: Absorption cross-section of a gold shell (diameter: 80 nm, thickness: 10 nm) compared to a solid gold sphere (diameter: 80 nm).*

***Resonance of a gold shell vis-à-vis HMCS***


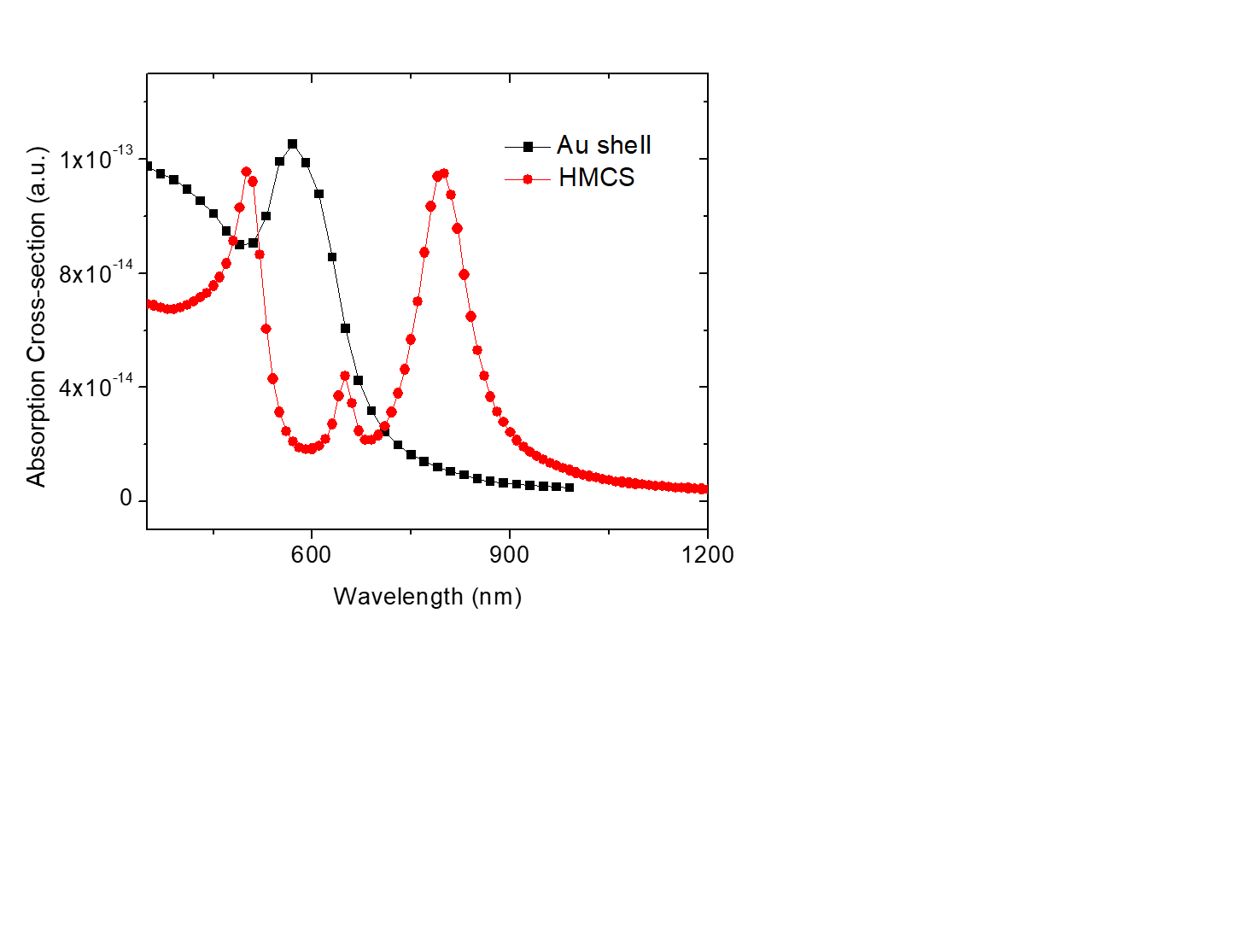


*Figure S5: Absorption cross-section of a gold shell (diameter: 180 nm, thickness: 10 nm) compared to a HMCS structure (Silver core - diameter 80 nm, dielectric shell - thickness 40 nm, gold shell - diameter 10 nm)*.

References:

1. [Palik, E. D. *Handbook of Optical Constants of Solids*. (Academic Press, 2012).](http://paperpile.com/b/mVBBkg/3qUZ)

2. [Johnson, P. B. & Christy, R. W. Optical Constants of the Noble Metals. *Physical Review B* **6**, 4370–4379 (1972).](http://paperpile.com/b/mVBBkg/cwXR)

3. [Rakić, A. D., Djurišić, A. B., Elazar, J. M. & Majewski, M. L. Optical properties of metallic films for vertical-cavity optoelectronic devices. *Applied Optics* **37**, 5271 (1998).](http://paperpile.com/b/mVBBkg/Z79X)

4. [Prodan, E. & Nordlander, P. Plasmon hybridization in spherical nanoparticles. *J. Chem. Phys.* **120**, 5444–5454 (2004).](http://paperpile.com/b/mVBBkg/Msmb)
